# Nanopowders of Yttria-Stabilized Zirconia Doped with Rare Earth Elements as Adsorbents of Humic Acids

**DOI:** 10.3390/ma12233915

**Published:** 2019-11-27

**Authors:** Małgorzata Suchanek, Ewa Niewiara, Katarzyna Wilkosz, Władysław W. Kubiak

**Affiliations:** Faculty of Materials Science and Ceramics, Department of Analytical Chemistry, AGH University of Science and Technology, Al. Mickiewicza 30, 30–059 Krakow, Poland; niewiara@agh.edu.pl (E.N.); katwilkosz@gmail.com (K.W.); kubiak@agh.edu.pl (W.W.K.)

**Keywords:** zirconia rare earth oxides, adsorption properties, humic acids

## Abstract

The aim of the investigations was to use, for the first time, zirconia nanopowders stabilized with yttria (YSZ) and rare element oxides (YSZ-Nd, YSZ-Gd) for removal of humic acids (HA) from aqueous solutions. Nanopowders were synthesized by means of hydrothermal crystallization and characterized using scanning electron microscope (SEM) with energy dispersive X-ray spectroscopy (EDX), X-ray diffraction (XRD), Fourier transform infrared (FT-IR) methods and analysis of zeta potential. The adsorption processes analysis was carried out in a series of experiments depending on: initial concentration of humic acids, contact time, pH and mass of the used adsorbent. It was found, that the YSZ-Nd exhibits strong and much higher effectiveness of HA adsorption than YSZ and YSZ-Gd. The HA adsorption rate reached 96.8% for YSZ-Nd dosage of 100 mg, pH 4, and 15 min reaction time and for HA initial concentration equal to 25 mg/L. According to the Langmuir model simulation, the maximum adsorption capacity of HA on YSZ-Nd at pH 4 was calculated to be 2.95 mg/g. Changes in the FT-IR spectra of YSZ-Nd confirmed humic acids’ adsorption on the tested nanopowders, by the presence of additional bands representing carboxylic, alcohol, carbonyl and amino groups in humic acid structure. These functional groups could represent humic acids binding on the YSZ, YSZ-Nd or YSZ-Gd surfaces.

## 1. Introduction

Humic substances constitute a group of natural organic matter present in soil, water reservoirs, sediments and natural water bodies. They are formed by decomposition of plants and animal remains. Humic substances can be divided into three components depending on their solubility: humic acids, fulvic acids and humin. Humic acids are not soluble in water below pH 2 but become soluble at higher pH. They belong to high molecular weight fraction and they are aromatic and aliphatic, what was referred in literature [1,2]. Fulvic acids are soluble and humin are insoluble in water at the entire pH range. Commercial humic acids are extracted from peat, coal and could be produced by fermentation using the empty fruit bunch of palm trees as a substrate. Humic acids could be produced through polymerization or condensation reactions in chemical synthesis. Humic acids form micelle-like structures, called pseudo-micelles in neutral to acidic conditions. The chemical composition of humic acids are different according to geographical origin, age, climate and biological conditions. Therefor characterization of those substances are difficult [3]. Humic acids (HA) have a complex structure, they are hydrophobic, heterogeneous and mostly aromatic. They contain mainly acidic functional groups such as carboxylic and phenolic, which induce negative charge in natural waters. In addition, humic acids contain carbonyl, hydroxyl, aldehyde acid and methoxyl functional groups and have a yellow to black color [4,5]. The presence of humic acids in water, especially in drinking water affects many quality parameters such as color, taste and odor. Humic acids contaminate membranes and ion-exchange resins. During the water treatment processes (disinfection) some carcinogenic products such as CHCl_3_ can be formed [6,7]. Humic acids are generally considered soluble in neutral to alkaline medium. This property varies with chemical composition, pH and ionic strength of those substances. In lower pH, phenolic and carboxylic groups are protonated and the repulsion of these negatively charged groups are minimalized, causing the molecule to adopt a coiled and compact structure. Upon increasing the pH, the functional groups are deprotonated and the repulsion of these groups causes the molecules to assume a stretched configuration. Furthermore, pH media affects the stability of aqueous suspensions of HA [3]. Humic acids form complexes with heavy metals and associations with organic pollutants. They may develop interactions with viruses and nanoparticles and affect transport of contaminants into the natural environment [8,9,10,11,12,13,14,15,16]. Hence, there is a need to eliminate HA from water despite their natural origin. Chemical coagulation, membrane separation and initial oxidation have been used successfully but these processes generate high operation costs. Adsorption is much more cost-effective process in water purification. Thus many low-cost adsorbents have been developed for organic matter removal. Previous researches have suggested that activated carbon [17,18], clays [5,19,20,21,22,23], biopolymers [4,24], iron oxide [25,26,27], magnetite [28], goethite [29], ZnO [30], graphite oxide [7], MgO nanoparticles [31] and SiO_2_ particles [32] can be proposed as adsorbents in order to remove humic acids from aqueous solutions. Nevertheless, effective humic acids’ adsorbents characterized by low production costs are still being sought. 

Pure zirconia crystallizes in three forms—cubic, tetragonal and monoclinic. The monoclinic polymorphs stabilize below 1160 °C. The cubic and tetragonal modification are stable from 2680 to 2370 °C and from 2370 to 1160 °C, respectively [33]. The high temperature zirconia polymorphs structure can be stabilized at room temperature by addition of various oxides such as magnesium oxide, calcium oxide or cerium oxide. Yttrium oxide is the most common component introduced into the zirconium structure, because it can be used as electrolyte in the solid oxide fuel cells (SOFC). Depending on the percentage of yttrium oxide in the structure, the tetragonal and monoclinic structure can be observed [34]. Unique properties of the yttria doped zirconia solid solution (YSZ) such as chemical and thermal resistance, high ionic conductivity [35,36] and optical properties [37] have been described in several publications. In addition, oxides of rare earth elements, such as Ln, Ce, Yb, Sm, Gd, Nd can be used to modify the structure of YSZ. The former studies showed the possibility of modification of YSZ up to 8 mol % of Nd or Gd. Such modifications cause improvement of mechanical and optical properties [37,38]. It should be stressed that yttria-stabilized zirconia (YSZ) and it is modifications (such as YSZ-Nd, YSZ-Gd) have never been used in the studies of adsorption processes. The powders of the ZrO_2_-Y_2_O_3_-Me_2_O_3_ system (Me = Sm, Nd, Gd, La) were manufactured by various methods such as co-precipitation and calcination, atmospheric plasma spraying [38,39,40,41] and by crystallization of the zirconia solid solution under hydrothermal conditions. The later process allows to obtain isometric crystallites of nanometric size [37,42]. 

The aim of the research was to demonstrate, for the first time, usefulness of the yttria-stabilized zirconia nanopowders (also doped with rare earth elements) in removal of humic acids (HA) from aqueous solutions. The nanopowders were prepared by the crystallization under hydrothermal conditions method and characterized in terms of their morphology, surface charge and HA adsorption properties. The adsorption efficiency was tested in the series of experiments, considering various initial parameters, that is, concentrations of humic acids, contact time, pH of the solutions and mass of the adsorbent. The desorption process was carried in distilled water. Additionally, the degree of HA adsorption on nanopowders was controlled by the FT-IR analysis. The low-cost production of yttria-stabilized zirconia (YSZ), zirconia stabilized with yttria and neodymium (YSZ-Nd), zirconia stabilized with yttria and gadolinium (YSZ-Gd) nanopowders, low-cost of adsorption process and high adsorption effectiveness allow successful usage of the above listed components in humic acids’ removal from water solutions. 

## 2. Materials and Methods 

Zirconium oxychloride octahydrate (ZrOCl∙8H_2_O), yttrium oxide (Y_2_O_3_), gadolinium nitrate hexahydrate and neodymium oxide were purchased from Sigma Aldrich and used as received for nanopowders synthesis of 3 mol % yttria-stabilized zirconia (YSZ), YSZ doped with Nd and YSZ doped with Gd. Chemicals such as HCl, NH_4_OH, NaOH used in synthesis and adsorption tests were of analytical grade. 

One g/L stock solution of humic acids (Sigma Aldrich, Steinheim, Germany) was prepared by dissolution of HA in the proper volume of double distilled water. 

### 2.1. Synthesis of YSZ, YSZ-Nd, YSZ-Gd Nanopowders

Three mol % yttria-doped zirconia nanopowder (YSZ) was prepared by the hydrothermal method. Zirconium oxychloride octahydrate and yttrium oxide were dissolved in nitric acid to obtain the ZrO_2_:Y_2_O_3_ ratio 97:3 mol %. The received solutions of zirconium and yttrium nitrates were introduced into ammonia water solution. The obtained gel was washed with distilled water to remove ammonium chloride and nitrate salts. The co-precipitated gel was hydro-thermally treated at 250 °C for 4 h under water vapor pressure and then was dried at 50 °C for 4 h. The Nd-doped YSZ (YSZ-Nd) and Gd-doped YSZ (YSZ-Gd) were synthesized according to the same procedure as YSZ but with the ZrO_2_:Y_2_O_3_:Nd_2_O_3_ (or Gd_2_O_3_) ratio equal 97:2:1 mol %. 

### 2.2. Characterization of the Synthesized Nanopowders

The surface area, total pore volume of all samples were measured by N_2_ adsorption using Brunauer-Emmett-Teller (BET) and Langmuir methods (ASAP 2010, Micromeritics, Norcross, USA. Microstructure and surface morphology of YSZ and its modification were observed under SEM microscope (FEI Nova NanoSem 200, Thermo Fisher Scientific, Hillsboro, Oregon, USA) with EDX analysis with an accelerating voltage 18 kV and magnification 20,000. The surface functional groups were analyzed by Fourier transform infrared spectroscopy (FT-IR) using KBr pellets and wavelengths range 4000–400 cm^−1^ (Bruker VERTEX 70v, FT-IR Spectrometer, Bruker Optics Inc., Billerica, MA USA). The phase composition and crystallite size of YSZ, YSZ-Nd and YSZ-Gd were investigated by the X-ray diffraction (XRD) technique with a Cu-Kα as the radiation source (X-ray Diffractometer Empyrean/PANanalytical, Almelo, The Netherlands) using Scherrer formula. The zeta potential of YSZ, YSZ-Nd and YSZ-Gd was measured using the Zeta potential analyzer (Zetasizer Nano-ZS, Malvern Panalytical Ltd, Malvern, United Kingdom). The pH of YSZ, YSZ-Nd or YSZ-Gd samples were measured as follows—0.1 g of nanopowder was mixed with 10ml of distilled water and shaken for 24 h at room temperature. The suspension pH was measured with a pH meter and a glass electrode (CPI 505, Elmatron, Zabrze, Poland). 

### 2.3. Study of Humic Acids Adsorption

The adsorption experiments were conducted at initial humic acids (HA) concentrations of 5, 10, 15, 20, 25, 30, 50, 70 mg/L, with contact times equal to 5, 10, 15, 30, 60, 90 and 120 min, at several pH values of 2, 4, 5, 6 and 7.5 and using various amount of adsorbent (50, 100, 200 and 500 mg) in 10 mL of double distilled water. The experiment were performed in temperature set to 20 °C. The pH of the suspensions was set using 0.1 M HCl and 0.1 M NaOH. The adsorption experiments were carried in the orbital-shaker at 100 rpm. After adsorption the suspension was separated by means of centrifugation at 13500 rpm. The supernatant was analyzed using the Ultraviolet-visible (UV-VIS) spectrophotometer (JascoV-630, JASCO Deutschland GmbH, Pfungstadt, Germany). The HA concentration was determined with a calibration curve drawn at 254 nm. The effect of HA removal was calculated by the Equation (1):

(1) $$\varepsilon_{ads}=\frac{C_{0}-C_{x}}{C_{0}}\times 100\%$$

where *C*_0_ and *C_x_* denote the initial and final HA concentration (mg/L), respectively; $\varepsilon_{ads}$ is the effectiveness of adsorption (%). The adsorbent separated from the suspension was then used in the desorption experiments performed in 10 mL of double distilled water. Experiments were conducted in the same orbital-shaker at 100 rpm for 15 min and then the suspension was centrifuged. The obtained solution was analyzed using the UV-VIS spectrophotometry. The effect of HA desorption was calculated by the Equation (2):

(2) $$\varepsilon_{des}=\frac{C_{des}}{C_{ads}}\times 100\%$$

where *C_des_* and *C_ads_* are the HA concentrations after desorption and adsorption, respectively; *ε_des_* is the effectiveness of desorption (%).

Nanopowders after the subsequent adsorption and desorption experiments were dried at 50 °C and subjected to FT-IR analysis.

## 3. Results and Discussion

### 3.1. Characterization of the Adsorbent

**Figure 1:**
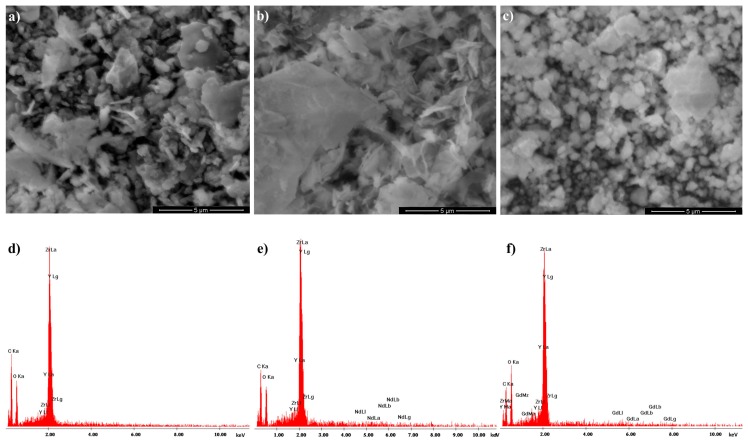
Scanning electron microscope (SEM) micrographs of (**a**) YSZ, (**b**) YSZ-Nd, (**c**) YSZ-Gd with energy dispersive X-ray (EDX) spectra of (**d**) YSZ, (**e**) YSZ-Nd, (**f**) YSZ-Gd.

By means of the BET it was found, that the specific surface area of the examined nanopowders were respectively YSZ 139.1 m^2^/g, YSZ-Nd 122.8 m^2^/g and YSZ-Gd 139.0 m^2^/g. The surface areas obtained from the Langmuir equation were accordingly 187.9 m^2^/g, 169.1 m^2^/g and 191.5 m^2^/g for YSZ, YSZ-Nd and YSZ-Gd. SEM images of the synthesized YSZ, YSZ-Nd and YSZ-Gd nanopowders are shown in Figure 1. As it can be seen, majority of particles were in the size range 50–500 nm, however in case of gadolinium containing nanopowders dimensions shift to smaller particles of cubic shapes. Nanopowders containing neodymium formation of larger particles of lamellar shape is favored. The EDX characterization of chemical composition confirmed the modification of YSZ structure by addition of Nd and Gd.

**Figure 2:**
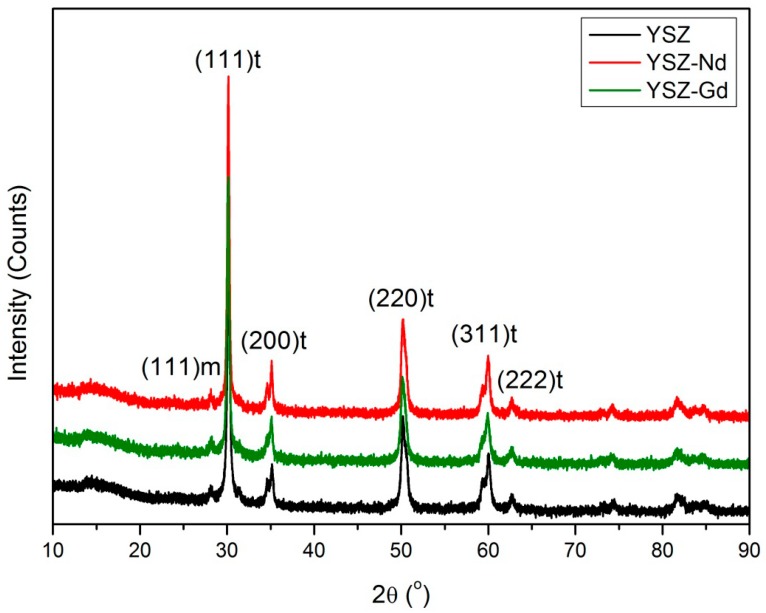
X-ray diffraction (XRD) patterns of YSZ, YSZ-Nd and YSZ-Gd.

Structure and the crystal phase composition of as-synthesized YSZ, YSZ-Nd and YSZ-Gd were characterized by the XRD pattern (Figure 2). The very intense and narrow peak at 2θ = 30.5° corresponds to the (111) planes of tetragonal phase. The distinguishing peaks for the tetragonal (t) phase of YSZ, YSZ-Nd and YSZ-Gd occurred at 2θ = 35°, 50.2°, 60.02° and 62.6° for (200), (220), (311) and (222) reflections, respectively. The peak at 2θ = 28.1° indicated the monoclinic (m) phase of YSZ, YSZ-Nd and YSZ-Gd. The nanopowder included mainly tetragonal phase with a small amount up to 15% of the monoclinic phase. Intensity of the peaks at 2θ = 28.1° and 30.5° indicated that YSZ-Nd had 1.2% of monoclinic phase and 98.8% of tetragonal phase comparing to YSZ and YSZ-Gd which had 9.2% and 13.9% of the monoclinic phase, respectively. The crystallite sizes calculated on the basis of the X-ray diffraction method from tetragonal phase line (011) broadening were 7 nm, 8.6 nm and 7.9 nm for the YSZ, YSZ-Nd and YSZ-Gd nanopowders, respectively.

**Figure 3:**
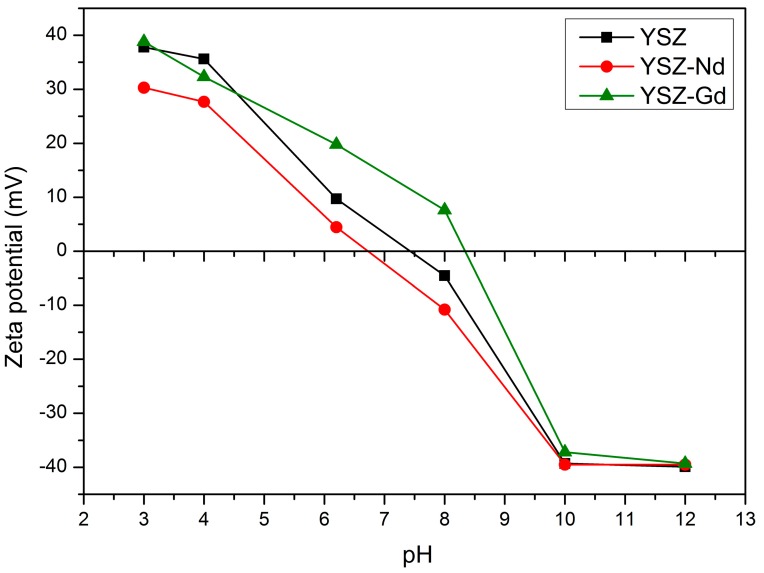
Zeta potential of YSZ, YSZ-Nd and YSZ-Gd as a function of pH in double distilled water.

Application of the synthetized nanopowders for humic acids adsorption in water require their use in suspension. Thus, their colloidal properties, such as zeta potential and its dependence on pH, are important. The zeta potential dependence on pH for the suspensions of YSZ, YSZ-Nd and YSZ-Gd are shown in Figure 3. 

It can be seen that undoped YSZ has point of zero charge at pH 7.6. Interesting is that doping with Gd and Nd shift the point of zero charge (pH_pzc_) in the opposite directions. YSZ-Nd shows the point of zero charge at pH below 7 whereas YSZ-Gd at pH 8.3.

### 3.2. Effect of pH on HA Adsorption on Nanopowders

**Figure 4:**
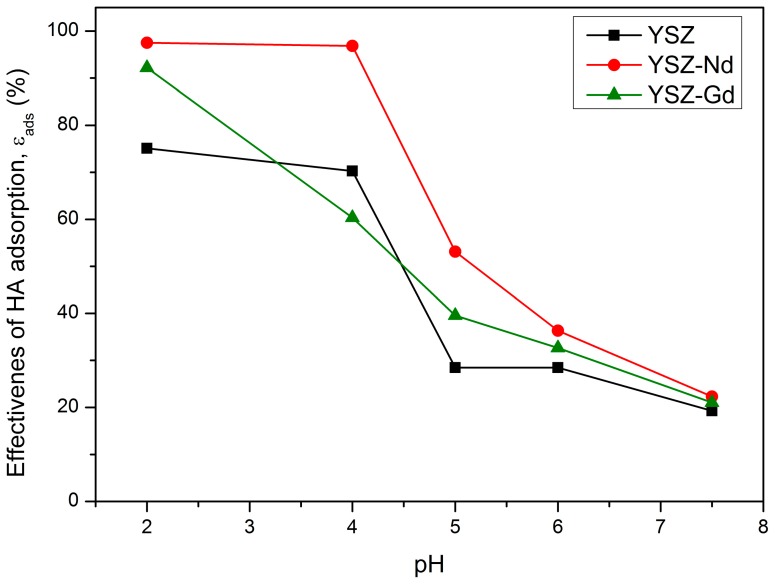
Effect of suspension pH on humic acid (HA) adsorption on YSZ, YSZ-Nd and YSZ-Gd (HA 25 mg/L, adsorbent 100 mg, contact time 15 min).

The influence of pH on humic acids adsorption was examined in the following conditions: pH ranged between 2 and 7.5, contact time was set to 15 min, humic acids concentrations was 25 mg/L and 100 mg portions of each nanopowder (YSZ, YSZ-Nd, YSZ-Gd) were used. It was observed that YSZ and modified nanopowders have a good adsorption capacity in wide pH range (Figure 4). With increase of the suspension pH from 2 to 7.5, efficiency of HA adsorption decreased. In acidic suspensions (pH 4) the YSZ, YSZ-Nd and YSZ-Gd nanopowders adsorbed 70.3%, 96.8% and 60.4% of humic acids, respectively. In alkaline pH (7.5) the results were 19.2%, 22.3% and 21.0%, respectively. Hence, the obtained results indicate that in the acidic solutions efficiency of humic acids adsorption is higher than in neutral and alkaline pH. The YSZ-Nd demonstrates the best sorption properties in comparison with YSZ and YSZ-Gd nanopowders. At pH values 2, 4, 5, 6 and 7.5, the effectiveness of HA adsorption on YSZ-Nd particles in the suspension was 97.5%, 96.8%, 53.1%, 36.3% and 22.3%, respectively. The experiment revealed that the optimal pH of the suspension, in respect to HA adsorption, was equal to 4. Similar results were reported for HA removal by the adsorbents such as chitosan-coated granules, iron oxides nanoparticles, magnetite nanoparticles, graphite oxide and bentonite [5,7,24,28].

When pH < pH_pzc_ (point of zero charge), the adsorbent should be cationic on its surface and exhibit tendency to attract anions. At pH > pH_pzc_, hydroxide groups are dominant and the adsorbent surface should be negatively charged and tend to attract cations. Humic acids contain several functional groups, which makes them negatively charged -COOH, -OH, -NH_2_, phenolic. The development of an electrical double layer on the adsorbent surface also influences the effectiveness of adsorption, changing polarity from positive to negative when pH changes from acidic to alkaline. Figure 3 shows that point of zero charge of YSZ, YSZ-Nd and YSZ-Gd were 7.4, 6.7 and 8.3, respectively. Thus, at low pH < 6.7, the surface charges of YSZ, YSZ-Nd and YSZ-Gd are positive, which allows the creation of an attracting force between the positive adsorbent surface and negative molecules of humic acids. That may be the reason of decrease of humic acids adsorption at higher pH. Moreover, the carboxylic groups of humic acids dissociate at pH value 4–6 and the phenolic groups at higher pH values, which explains lower adsorption effectiveness at higher pH [7]. 

### 3.3. Effect of Adsorbent Dose 

**Figure 5:**
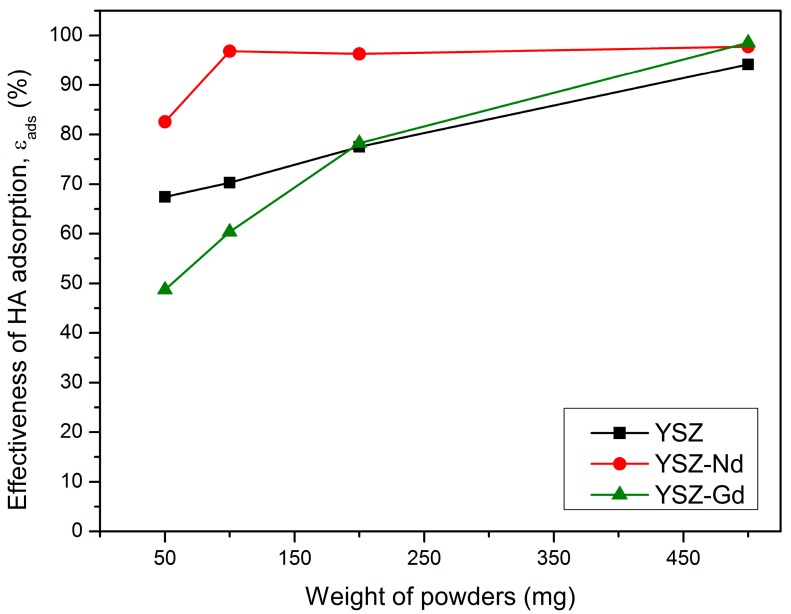
Effect of YSZ, YSZ-Nd and YSZ-Gd dosage on HA adsorption (HA 25 mg/L, pH 4, contact time 15 min).

The influence of adsorbent amount (50, 100, 200 and 500 mg) on humic acids adsorption was tested for each YSZ, YSZ-Nd and YSZ-Gd nanopowder, at a contact time of 15 min. The humic acid concentrations were 25 mg/L and the suspension pH was set to 4. It was observed that YSZ nanopowder and its modifications have a good adsorption ability in the tested amount range (Figure 5). Increasing the mass of the adsorbent lead to increasing efficiency of HA adsorption. The study showed that the application of 50 mg of nanopowder—YSZ, YSZ-Nd and YSZ-Gd adsorbed 67.4%, 82.6% and 48.7% of the humic acids, respectively. Furthermore, HA adsorption was the highest for 500mg of the adsorbent. The YSZ-Nd had better adsorption properties in respect to humic acids, than YSZ and YSZ-Gd nanopowders. For the tested YSZ-Nd nanopowder amounts 50, 100, 200 and 500 mg, effectiveness of adsorption was 82.6%, 96.8%, 96.3% and 97.7%, respectively. Increase of the adsorption efficiency with the increase of the adsorbent dose can be attributed to higher surface area and a higher number of active sites of the adsorbent. As a result, more molecules of HA can be bounded to YSZ, YSZ-Nd or YSZ-Gd. Thus, the higher the rate of HA adsorption would be. It was found that 100 mg portions of nanopowder were the optimal amount for effective adsorption of HA. 

### 3.4. Effect of Contact Time

**Figure 6:**
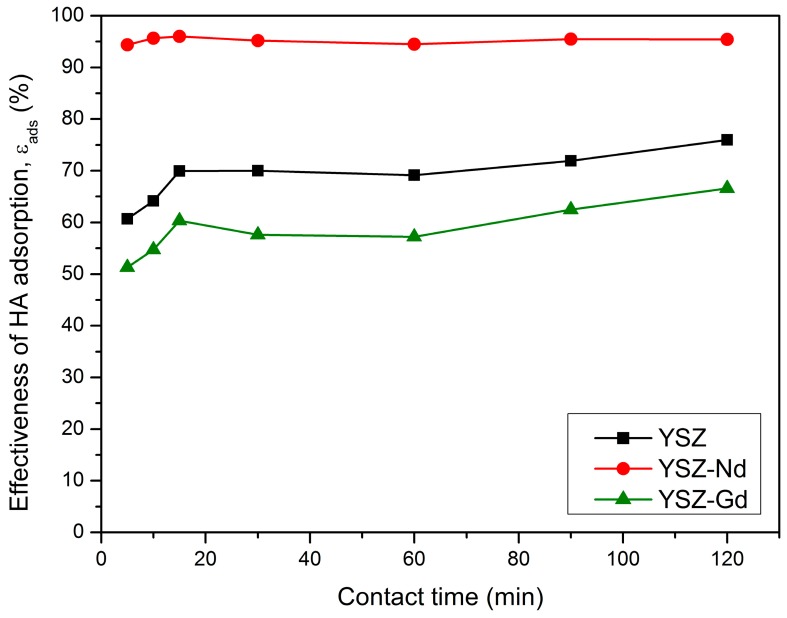
Effect of contact time on the HA adsorption on YSZ, YSZ-Nd and YSZ-Gd (HA 25 mg/L, adsorbent 100 mg, pH 4).

The effect of contact time was investigated in the range of 5–120 min with the initial HA concentrations of 25 mg/L, pH value set to 4 and 100 mg of each YSZ, YSZ-Nd and YSZ-Gd nanopowders in room temperature. As shown in Figure 6, for the YSZ and YSZ-Gd nanopowders the HA adsorption efficiency increased with the increase of contact time during the first 15 min. Later, the adsorption efficiency remained nearly constant. The effectiveness of HA adsorption on YSZ-Nd was stable in the whole range of contact time and reached from 94.4% to 96.0%. After 15 min of adsorption on YSZ, YSZ-Nd and YSZ-Gd, the effectiveness of HA adsorption was 69.9%, 96.0% and 60.4%, respectively. The results of these experiments determined the optimal contact time as 15 min.

Adsorption of HA was rapid at the beginning of the process because there was a large number of active sites on the surface of the adsorbent. Over time, the number of active sites decreased as the molecules of humic acids occupied those sites and the adsorption could occur in the inner layer with a slower adsorption rate. Also, because of increase of the repulsive forces between the adsorbed molecules of humic acids on the surface of the adsorbent, the rate of adsorption decreases. A similar relationship was observed on bentonite and montmorillonite [23], ZnO [30], graphite and graphite oxide [7] and magnetic multi-walled carbon nanotubes modified with polyaluminium chloride [43]. 

### 3.5. Effect of Initial Concentration of Humic Acids 

**Figure 7:**
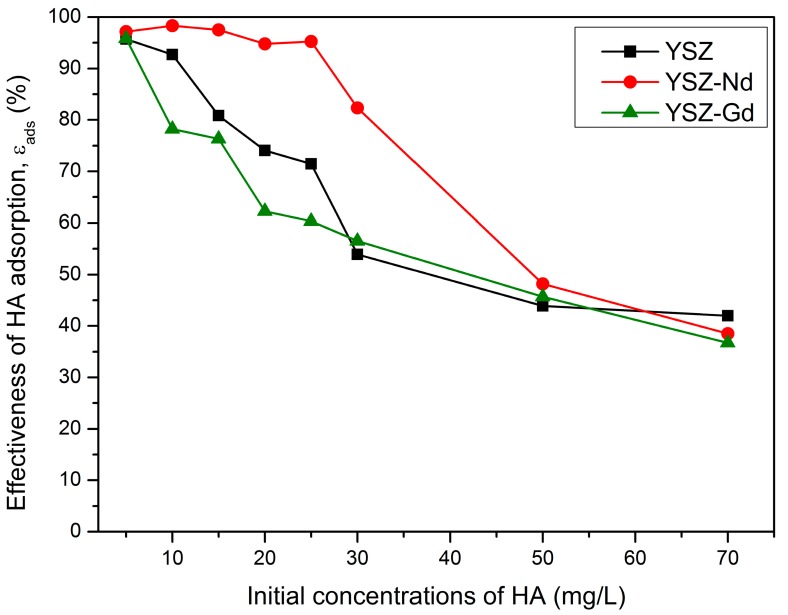
Effect of HA initial concentrations on the HA adsorption (adsorbent 100 mg, pH 4, contact time 15 min).

Figure 7 shows the effect of initial concentrations of HA on their adsorption on YSZ, YSZ-Nd or YSZ-Gd. With increase of the initial concentrations of HA, effectiveness of its removal from the solution decreased. The effect was tested using 100 mg of each of the studied nanopowders, at contact time 15 min, pH value of 4 and humic acids concentrations of 5, 10, 15, 20, 25, 30, 50 and 70 mg/L. When humic acids concentrations were below 25 mg/L, the HA adsorption efficiency was over 95% on the YSZ-Nd adsorption process, while HA initial concentrations were above 25 mg/L, the HA adsorption efficiency decreased sharply. The similar results were observed on multi-walled carbon nanotubes [43]. The reason of this dependence is that adsorption surface is large at lower HA concentrations and decreases as the concentrations increases and repulsive forces between molecules occur [30]. 

### 3.6. Adsorption Isotherms

**Figure 8:**
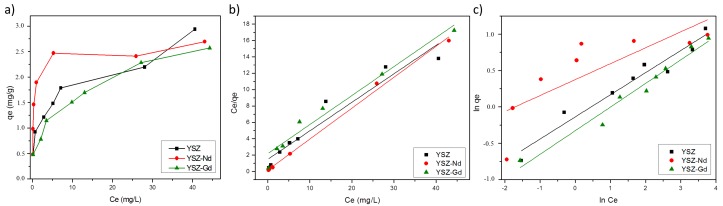
The adsorption isotherms (**a**) and comparison of the experimental q_e_ values with those by (**b**) Langmuir isotherm model, (**c**) Freundlich isotherm model for the adsorption of HA on YSZ, YSZ-Nd, YSZ-Gd for various initial concentrations of HA.

The equilibrium adsorption isotherm was used to understand the mechanism of adsorption in the studied system. The adsorption isotherm describes the relation between the adsorbent and the optimal amount of adsorbent. Figure 8a shows the adsorption isotherms of HA on YSZ, YSZ-Nd and YSZ-Gd nanopowders. The YSZ-Nd nanopowder exhibits higher adsorption than YSZ and YSZ-Gd. 

From the available isotherm models Langmuir and Freundlich isotherm were selected for the tests herein, using 100 mg of YSZ, YSZ-Nd or YSZ-Gd nanopowders, humic acids concentrations of 5, 10, 15, 20, 25, 30, 50 and 70 mg/L and pH 4. 

#### 3.6.1. Langmuir Isotherm

The Langmuir isotherm assumes the existence of a single coating layer placed on the adsorbent surface and that the attraction between molecules decreases as getting further from the adsorbent surface. The Langmuir equation is expressed as:

(3) $$q_{e}=\frac{q_{m}K_{L}C_{e}}{1+K_{L}C_{e}},$$

where *q_e_* is the amount of HA adsorbed per unit weight of YSZ, YSZ-Nd or YSZ-Gd at the equilibrium concentration (mg/g), *C_e_* is the equilibrium HA concentration in the solution (mg/L), *q_m_* is the amount of solute adsorbed per weight of adsorbent at monolayer coverage (mg/g) and *K_L_* is the Langmuir adsorption equilibrium constant related to the adsorption energy. The plots *C_e_*/*q_e_* versus *C_e_* for HA adsorption on YSZ, YSZ-Nd and YSZ-Gd for initial HA concentrations ranging from 5 to 70 mg/L were drawn (Figure 8b). The values of *q_m_* and *K_L_* were determined from the slope and intercept of the *C_e_*/*q_e_* versus *C_e_* plots and are shown in Table 1. 

#### 3.6.2. Freundlich Isotherm

The Freundlich model is based on the assumption that the adsorbent has a heterogeneous surface composed of different classes of adsorption sites and its usually applied to multilayer adsorption processes. Freundlich equation expresses as:

(4) $$\ln q_{e}=\ln K_{F}+n\ln C_{e},$$

where *K_F_* and *n* are the Freundlich constants (L/mg) representing the capacity and the intensity of adsorption. The data obtained from the HA adsorption on YSZ, YSZ-Nd and YSZ-Gd were fitted to the Freundlich model by plotting ln*q_e_* versus ln*C_e_* (Figure 8c). The values of K_F_ and n were determined and listed in Table 1. 

The results showed that the Langmuir isotherm produced slightly better fitting in terms of regression coefficient and it is well correlated with the experimental data of YSZ-Nd and YSZ-Gd nanopowders (R^2^ > 0.97). The Freundlich model is also correlated with the experimental data of YSZ-Gd and YSZ. Based on the Langmuir isotherm, the maxima of HA adsorption on YSZ, YSZ-Nd and YSZ-Gd were 2.87, 2.95 and 2.82 mg/g, respectively. Similarly, Hartono et al. in Reference [7] showed that adsorption of humic acids on graphite and graphite oxide was more similar to that in the Langmuir isotherm than the Freundlich one. Zhang et al. [24] published that adsorption of humic acids on chitosan granules was 0.41 mg/g, while Oskoei et al. [30] presented that removal of humic acids by nano-ZnO was 0.82 mg/g. However, Gang Xue et al. [44] reported the adsorption of humic acids on TiO_2_ activated carbon as 1 mg/g, which is lower than the nanopowders proposed in this work. 

### 3.7. Desorption of HA

**Figure 9:**
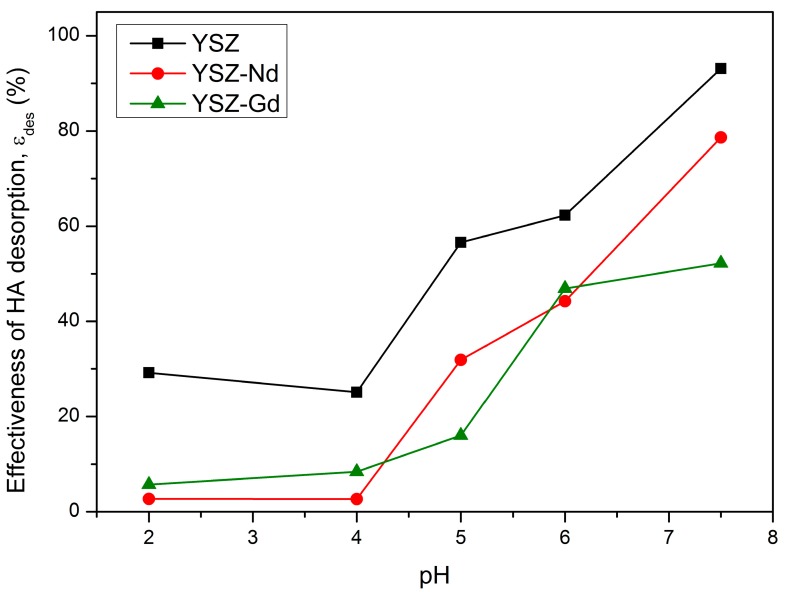
Effect of suspension pH on HA desorption from YSZ, YSZ-Nd and YSZ-Gd.

To investigate desorption of HA from YSZ, YSZ-Nd and YSZ-Gd in the pH range between 2 and 7.5 at contact time 15 min double distilled water was used. It was observed that with an increase of the suspension pH, the desorption efficiency increased (Figure 9). 

At pH 4, 29.2%, 2.7% and 8.4% of HA were desorbed from YSZ, YSZ-Nd and YSZ-Gd, respectively. From pH 2 to pH 7.5 the desorption efficiency increased from 2.7% to 78.7% for YSZ-Nd. The results have also revealed that the highest adsorption efficiency corresponds to the lowest desorption efficiency. That indicates that humic acids are permanently attached to the surface of the adsorbent in acidic solution. In alkaline solution however, humic acids can easily be removed from the adsorbent surface. Similar correlation was observed in case of HA desorption from YSZ and YSZ-Gd nanopowders. The addition of neodymium to the YSZ structure results in stronger binding of humic acids on its surface compared to YSZ.

### 3.8. FT-IR Analysis

**Figure 10:**
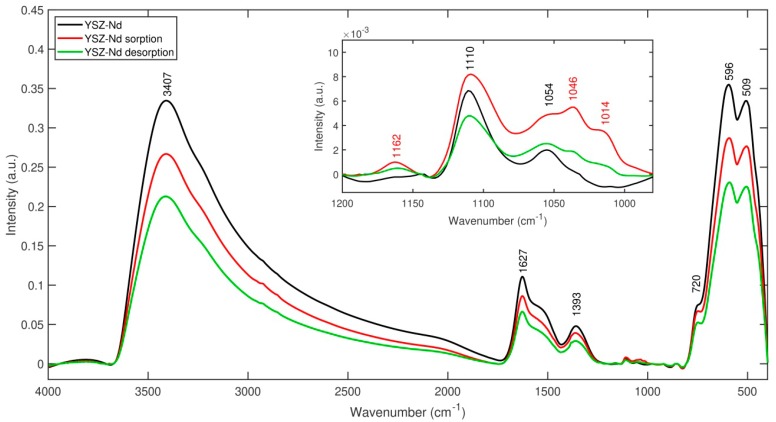
FT-IR spectra of YSZ-Nd (black), YSZ-Nd after HA adsorption (red), YSZ-Nd after HA desorption (green).

FT-IR spectroscopy of YSZ-Nd, YSZ-Nd after HA adsorption and YSZ-Nd after HA desorption was performed to confirm HA adsorption and was presented in Figure 10. As shown, there is a characteristic broad spectra between 3000 and 3600 cm^−1^ with maximum at 3407 cm^−1^ due to the stretching vibrations of structural OH groups, which is associated with carboxylates, phenols and alcohols [21]. The bands related to carboxylate asymmetric and symmetric stretching modes are observed at 1628 cm^−1^ and 1393 cm^−1^. The band below 800 cm^−1^ is assigned to metal oxygen stretching mode from chelating of ammonia and metallic ions (Zr, Y, Nd) [45]. Differences in the FT-IR spectra before and after HA adsorption or desorption were observed in range 1200–980 cm^−1^. The band appeared at 1162 cm^−1^ after HA adsorption on YSZ-Nd and was attributed to C-O stretching vibration of carboxylic groups and it was less intensive after desorption. The strong absorption band at 1125–1085 cm^−1^ was due to the secondary alcohol C-OH stretching. The appearance of additional band at 1046 cm^−1^ on YSZ-Nd after HA adsorption came mainly from the C-O functional groups of HA, which were observed by Song et al. [46]. The enhancement of the band at 1046 cm^−1^ after HA adsorption and its weakening after HA desorption confirmed that humic acids were bounded to YSZ-Nd and later desorbed. The absorption band noticed around 1014 cm^−1^, resulted from the presence of hydride ion [47]. However, the absorption bands in the range 1100–1000 cm^−1^ after HA adsorption on YSZ-Nd were more insensitive than after HA desorption and could be identified as the carbonyl C-O-C stretching, which was observed in the structure of humic acids as reported by Shaker and coworkers [21]. The difference of intensity band at 720 cm^−1^ after adsorption and desorption can be attributed to N-H bending out of plane, which corresponded to the presence of N-H bond from humic acids. The appearance of additional bands in the range 720–1640 cm^−1^, after HA adsorption and desorption, confirmed the presence of carboxyl, alcohols, carbonyl and phenols groups in the structure of YSZ-Nd proving that humic acids were adsorbed on the nanopowder. Similar FT-IR spectra and its differences were observed for YSZ and YSZ-Gd but less intensive bands after adsorption and desorption were observed, which confirmed that less HA adsorption on YSZ and YSZ-Gd comparing to YSZ-Nd.

## 4. Conclusions

In this study, the YSZ, YSZ-Nd and YSZ-Gd nanopowders were synthesized by crystallization under hydrothermal conditions and for the first time were applied for humic acids removal from aqueous solutions. Nanopowders were characterized in terms of their morphology, surface charge and HA adsorption properties. The processes were conducted at various initial concentrations of humic acids, contact times, pH of the suspension and mass of the adsorbent in a series of experiments. The desorption process was studied in distilled water. Supernatants were analyzed by UV-VIS spectrophotometer. HA concentrations were determined with calibrations curve drawn at 254 nm. Furthermore, the degree of HA adsorption on nanopowders was verified by FT-IR analysis. The addition of 1% mol. Nd_2_O_3_ to the YSZ structure significantly improved the material’s properties with respect to humic acids adsorption, while the addition of 1% mol. Gd_2_O_3_ decreased these properties. The YSZ-Nd nanopowder showed the best humic acids adsorption efficiency (96.8%) compared to YSZ (70.3%) and YSZ-Gd (60.4%). The improvement of YSZ-Nd adsorption properties might result from the percentage of crystal phase, because this nanopowder mainly contained a tetragonal phase with 1.2% of monoclinic phase content. The YSZ and YSZ-Gd nanopowders had higher content of monoclinic phase. Analysis of the adsorption conditions indicated that the best adsorption effectiveness in the solution containing 25 mg/L of humic acids was observed at pH 4, contact time 15 min and 100 mg of the adsorbent. The equilibrium data have been explored by Freundlich and Langmuir models. The results showed that the experimental data could be correlated with the Langmuir and Freundlich models. The Langmuir model showed better results for YSZ-Nd and the Freudlich one for YSZ and YSZ-Gd. Maxima of adsorption capacity of YSZ, YSZ-Nd and YSZ-Gd nanopowders were higher than on chitosan granules, nano-ZnO or TiO_2_ activated carbon. The desorption process confirmed the strong binding of humic acids to the nanopowder’s surface. However, desorption should be carried out in a strongly alkaline solution to regenerate the nanopowder. Changes in the FT-IR spectra for YSZ-Nd comparing to nanopowder after humic acids adsorption or desorption confirmed humic acids adsorption on the nanopowder’s surface. The additional functional groups represented carboxylic, alcohol, carbonyl and amino, presented in humic acid structure and they could be responsible for humic acids binding on YSZ, YSZ-Nd or YSZ-Gd surfaces. 

The proposed materials, YSZ, YSZ-Nd and YSZ-Gd, can be used for the effective removal of humic acids from the treated aqueous solution.

## Figures and Tables

**Table 1:** Parameters of the adsorption isotherms of YSZ, YSZ-Nd and YSZ-Gd.

Adsorbent	Langmuir Isotherm			Freundlich Isotherm		
	q_max_(mg/g)	K_L_ (L/mg)	R^2^	K_F_	n	R^2^
YSZ	2.87	0.234	0.934	0.872	3.289	0.951
YSZ-Nd	2.95	1.990	0.997	1.456	4.570	0.643
YSZ-Gd	2.82	0.162	0.967	0.722	3.081	0.971
